# Case Report: Explantation of A Binkhorst Iridocapsular Lens >30 Years After Implantation in an Eye With Pseudoexfoliation Syndrome

**DOI:** 10.1097/MD.0000000000001444

**Published:** 2015-08-28

**Authors:** Adriano Guarnieri, Javier Moreno-Montañés, Alfonso L. Sabater

**Affiliations:** From the Department of Ophthalmology, Clínica Universidad de Navarra, Pamplona, Spain.

## Abstract

An 86-year-old man with a Binkhorst 2-loop intraocular lens (IOL) that was implanted in the pupillary sphincter 33 years earlier was examined. The pupil of the implanted eye with the Binkhorst IOL was irregular and the eye had pseudoexfoliation (PEX) syndrome. Pupillary erosion resulted from rubbing of the IOL edge against the pupillary sphincter with PEX material. The IOL was removed because of visual distortion and intense pseudophakodonesis. Gross and light microscopic analyses showed no irido-fibro-lenticular adhesions over the lens or fragments of iris tissue attached to the lens. Scanning electron microscopy showed several pores of different sizes. No inflammatory cells were present, suggesting that the IOL was well tolerated.

The case suggested that the pupillary ruff was not a good location for implantation of an IOL in an eye with PEX. Caution is recommended before implanting or suturing an IOL close to the pupillary border in eyes with PEX during cataract surgery.

## INTRODUCTION

Binkhorst was an early advocate of iris-supported intraocular lenses (IOLs).^1^ He occasionally implanted his 4-loop iris-fixated lens after extracapsular cataract extraction (ECCE) rather than intracapsular cataract extraction, which prompted him to modify his iris-clip lens for implantation after ECCE to decrease the chafing against uveal tissues and the incidence of complications such as inflammation.^2^ In some cases, the IOL optic became dislocated behind the pupil, which resulted in immersion of the entire IOL in the capsular bag. Binkhorst's leadership in advocating the change to ECCE and the introduction of his 2-loop iridocapsular IOL were important advances in IOL design and mode of fixation, because these innovations led to modern capsular (in-the-bag) fixation of posterior chamber IOLs.^3^

We recently explanted and analyzed a Binkhorst 2-loop iridocapsular IOL that eroded the pupil in an eye with pseudoexfoliation (PEX) syndrome, a common age-related disorder affecting intraocular and extraocular tissues, characterized by the production and accumulation of an abnormal PEX fibrillar material. This IOL had been implanted for >30 years. To our knowledge, this is the longest follow-up of this obsolete IOL model that was explanted in a living patient with PEX syndrome.

## METHODS

**FIGURE 1:**
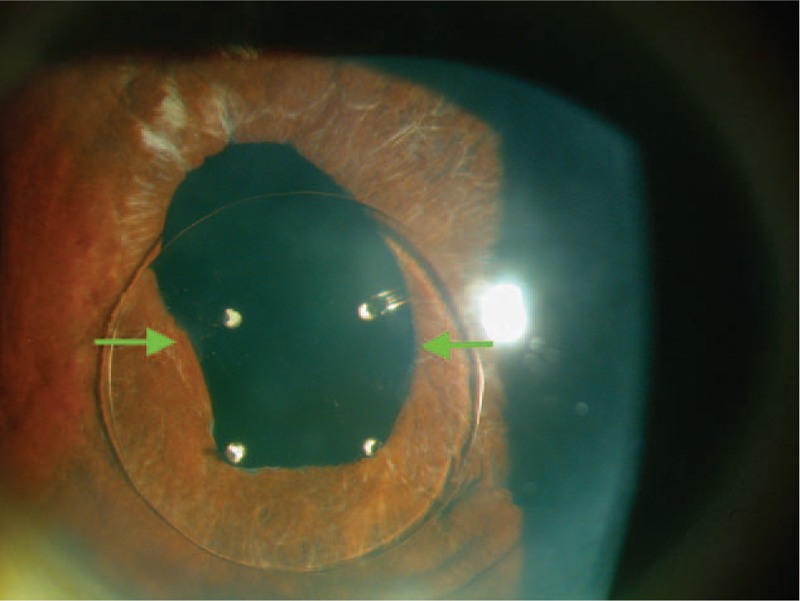
Biomicroscopy examination of the right eye shows a quiet anterior chamber, clear transparent cornea, irregular pupil with some iris atrophy, and downward decentration of the Binkhorst 2-loop iridocapsular intraocular lens (IOL). Some pseudoexfoliative material is seen at the pupillary border (green arrows). The inferior haptics drag the pupil down whereas the superior haptics are away from the pupillary border. The superior pupillary area is uncovered as the IOL is progressively decentered downward.

**FIGURE 2:**
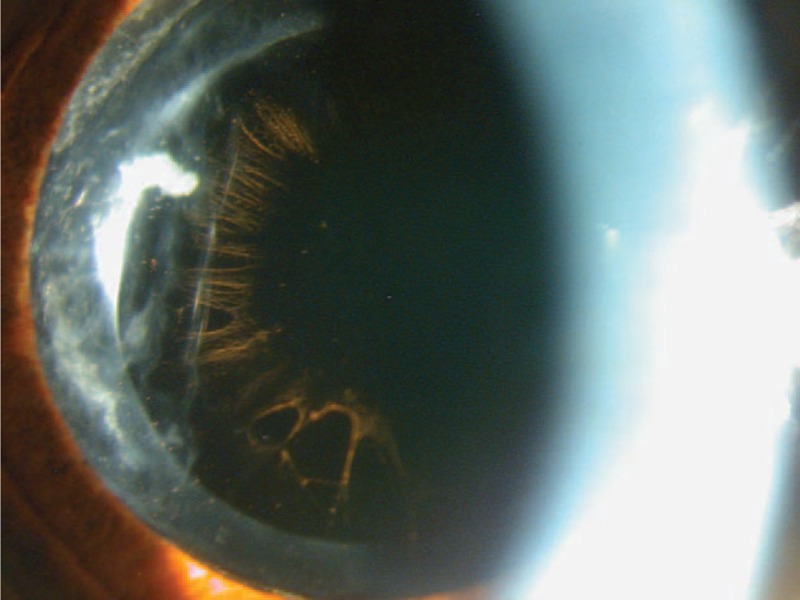
The fellow eye (left eye) with an in-the-bag intraocular lens, with pseudoexfoliative material and characteristic radial pattern pigmentation over the optic is seen with a dilated pupil.

An 86-year-old man presented for an ophthalmologic examination in May 2013 with the complaint of visual distortion of several months duration in his right eye. He did not report previous trauma, redness, or pain in either eye. He had undergone previous cataract surgery in both the eyes, 7 years before examination in the left eye and 33 years earlier in the right eye. The best-corrected visual acuity (BCVA) at examination was 20/40 in the right eye (−4.00 −4.50 × 130°) and 20/20 (plano) in the left eye. At the time of the examination, anisocoria was noted with an irregularly shaped pupil in the right eye (Figure 1). The patient had an iridocapsular IOL with the optic over the pupil and the haptics behind the iris. The inferior haptics were eroding the pupillary ruff, and the superior haptics were separated from the pupillary border. Intense pseudophakodonesis, atrophic signs in the iris sphincter, and PEX material at the iris edge was seen (Figure 1). There was a transparent cornea without inflammatory reaction in the anterior chamber. An irregular pupil partially covered by the IOL optic was observed, which explained the visual symptoms. Specular microscopy showed a normal endothelial pattern (cell count, 1986 cells/mm^2^) in the right eye. The fellow eye had an in-the-bag IOL, with PEX material over the IOL and radial pattern pigmentation seen through a dilated pupil (Figure 2). The posterior segments were normal in both the eyes. The intraocular pressure (IOP) values were 14 mm Hg in the right eye and 12 mm Hg in the left eye without medication.

**FIGURE 3:**
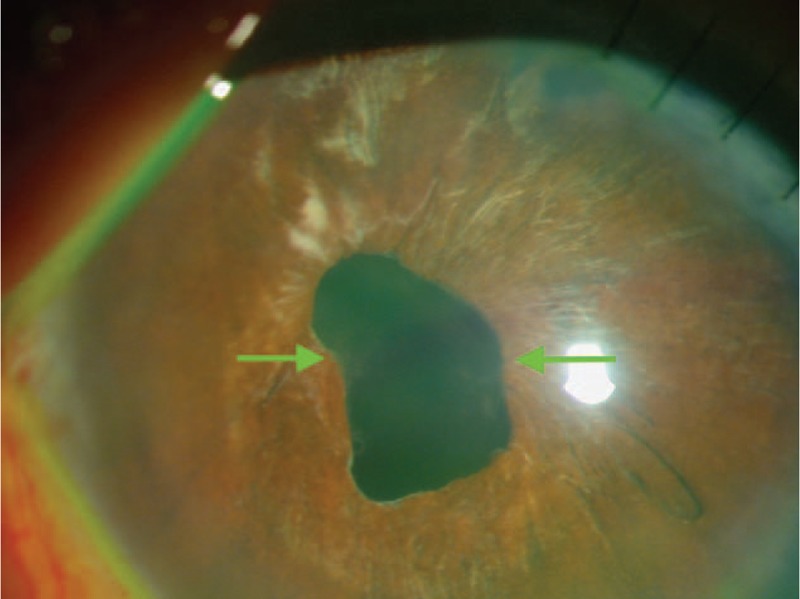
The implanted intraocular lens completely covers the pupil, which remained square and unresponsive to light. The green arrows indicate the points where the inferior haptics began to erode and change the pupillary size and shape.

The IOL instability in the right eye prompted us to recommend an IOL exchange to the patient. The patient gave informed consent regarding the surgery and publication of his medical records with educational interest. In the surgery suite, when the patient was face-up in the prone position, the IOL was centered, indicating the absence of adhesions between the iris and the IOL. A 6-mm clear corneal incision was made and the IOL was easily explanted without vitreous traction or any other intraoperative complication. The haptics did not require cutting to remove the IOL from the anterior chamber. A monofocal CZ70BD IOL (Alcon Laboratories, Fort Worth, TX) was sutured into the sulcus using a 9-0 prolene suture. One month postoperatively, the implanted IOL completely covered the pupil, which remained square and unresponsive to light (Figure 3). The BCVA after IOL exchange was 20/25 (+1.00 − 4.00 × 140°). No other complications developed 6 months postoperatively. The explanted Binkhorst 2-loop iridocapsular IOL was analyzed by optical and electron microscopy.

## RESULTS

**FIGURE 4:**
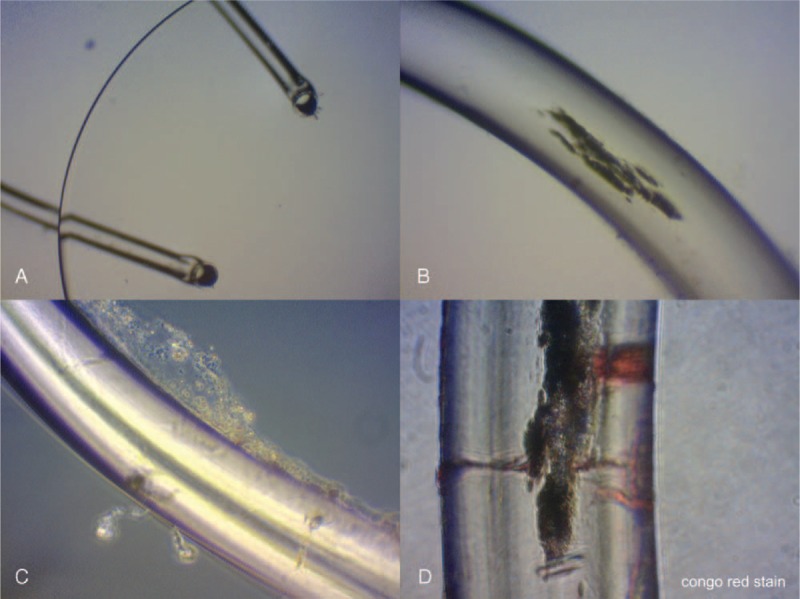
Phase contrast photomicrographs of the explanted Binkhorst 2-loop intraocular lens (IOL). (A) One of the 2-loop haptic insertions in the transparent optic of the lens is seen (original magnification, ×100). (B) Iris pigment is seen on the surface of the loops (original magnification, ×200). (C) Fibrotic and transparent material adheres to the border of the loops (original magnification, ×200). (D) Collagen material surrounding the iris pigment is seen on the surface of the loops (Congo red, original magnification, ×400).

**FIGURE 5:**
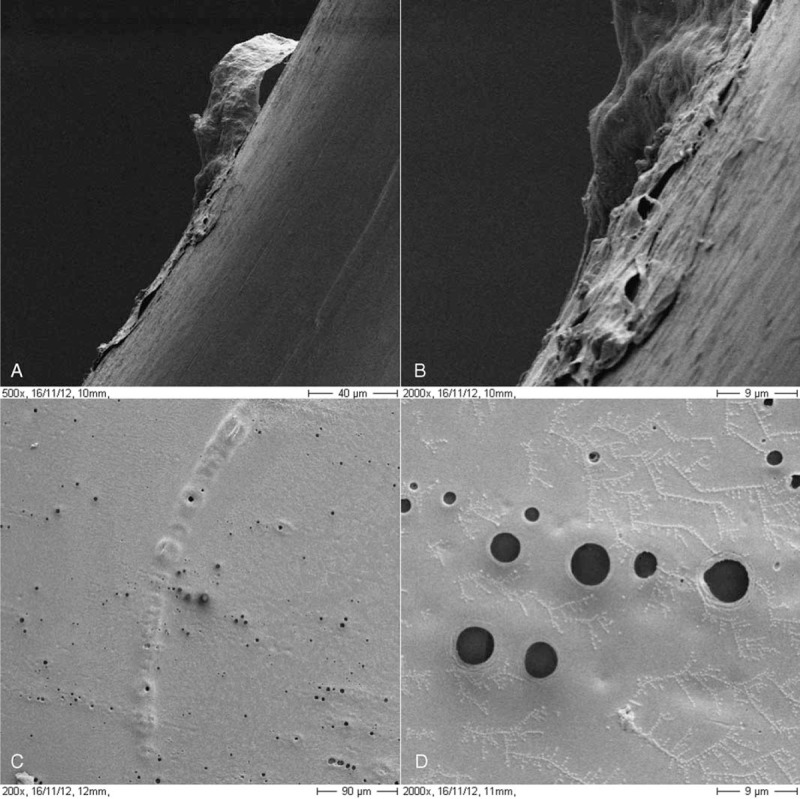
Scanning electron micrograph of the explanted Binkhorst 2-loop intraocular lens (IOL). (A) A fibrillary substance adheres to the haptic of the IOL (×500 magnification). (B) A fibrillary substance adheres to the haptic of the IOL (×2000 magnification). (C) Several holes are seen in the IOL optic (×200 magnification). (D) The sizes of the holes in the IOL optics vary in diameter from 1 to 9 μm (original magnification, ×2000).

Gross examination showed no irido-fibro-lenticular adhesions over the IOL or haptics, as there were no fragments of iris tissue attached to the IOL. Phase contrast photomicrographs (Figure 4) showed iris pigment on the surface of the loops and fibrotic and transparent material adhering to the border of the loops. A commercially available scanning electron microscope (Zeiss DSM 940A, Oberkochen, Germany) was used to analyze the IOL optic and haptics (Figure 5). Several pores of different sizes (<5 μm) were on the lens surface and nonspecific deposits were observed over the IOL haptics. We hypothesize that these pores had some kind of relationship with the explanted IOL being processed for evaluation. Finally, no inflammatory cells were seen with hematoxylin and eosin staining.

## DISCUSSION

The concept behind iris-fixated IOLs was avoidance of IOL dislocation, a major complication of the first posterior chamber IOLs, and corneal decompensation, the most important complication associated with anterior chamber IOLs at that time. In the current case, a Binkhorst 2-loop iridocapsular IOL was well tolerated in the anterior chamber for >30 years but later affected the pupil and became decentered. This eye had atrophy of the pupillary ruff characteristic of the PEX syndrome,^4^ which caused erosion of the iris by the IOL over the long term with subsequent IOL decentration. In our opinion, it is unlikely that the weight of the Binkhorst 2-loop lens (about 1 mg, similar to modern IOLs) caused the downward decentration, because the IOL remained in place for such a long time. Iris chafing and erosion are complications of iris-supported lenses, where excessive contact or rubbing erodes the iris and disrupts the blood-aqueous barrier causing inflammation, fibrosis, and erosion, which could lead to corneal decompensation or pigmentary glaucoma in addition to IOL dislocation. Fortunately, the endothelial pattern was normal for an 86-year-old man and the IOP was within normal values. The absence of synechiae during IOL removal, and morphologic analysis of the IOL, suggested that there were no significant intraocular inflammatory episodes in the right eye. These findings supported the experience that polymethylmethacrylate is well tolerated over the long term^5^ and suggested that the IOL was correctly implanted with both loops inside the capsular bag.^6^ The PEX material was found in the pupillary border associated with pupillary ruff atrophy, both characteristic signs of the PEX syndrome.^4^ The PEX material also was found on the IOL surface in the left eye, which had been implanted more recently, suggesting that the PEX syndrome developed late and therefore the IOL was stable on the pupil for many years.

The PEX syndrome is the most important risk factor for late IOL dislocation in the capsular bag.^7^ Surgeons must be aware of this condition when following a patient with a dislocated IOL. Although different approaches for secondary IOL implantation have similar results regarding visual acuity or endothelial complications,^8^ we recommend fixation of the IOL to the sulcus to avoid iris-related problems in advanced PEX syndrome. The current case is particularly interesting as few Binkhorst IOLs had been explanted from a living patient. The clinical findings strongly suggested that IOL subluxation was not related to its design but rather to pathological changes in the iris secondary to the PEX syndrome. Finally, although the disruption of the blood-aqueous barrier is a sign of the PEX syndrome, it is important that no inflammatory deposits were found on the IOL, although the IOL eroded the pupillary ruff and the IOL was unstable with intense pseudophakodonesis.

In conclusion, this IOL was not associated with an inflammatory reaction or deposits on the IOL after >30 years of implantation, although the blood-aqueous barrier does break down in eyes with PEX syndrome. This case also suggested that the pupillary sphincter is not an appropriate location for fixation of an IOL in an eye with PEX. Particular caution must be taken when a 3-piece IOL is sutured to the back of the iris or an iris-claw aphakic IOL is fixated near the pupillary sphincter in eyes with PEX syndrome.
